# Morphology engineering of silicon nanoparticles for better performance in Li-ion battery anodes†

**DOI:** 10.1039/d0na00770f

**Published:** 2020-10-13

**Authors:** Samson Y. Lai, Jan Petter Mæhlen, Thomas J. Preston, Marte O. Skare, Marius U. Nagell, Asbjørn Ulvestad, Daniel Lemordant, Alexey Y. Koposov

**Affiliations:** Department for Neutron Materials Characterization, Institute for Energy Technology (IFE) Instituttveien 18 NO-2007 Kjeller Norway; Department of Battery Technology, Institute for Energy Technology (IFE) Instituttveien 18 NO-2007 Kjeller Norway; PCM2E (EA6299) University of Tours, Faculté des Sciences et Techniques Bât. J, Parc de Grandmont 37200 Tours France; Centre for Materials Science and Nanotechnology, Department of Chemistry, University of Oslo PO Box 1033 Blindern Oslo N-0315 Norway alexey.koposov@kjemi.uio.no

## Abstract

Amorphous silicon nanoparticles were synthesized through pyrolysis of silane gas at temperatures ranging from 575 to 675 °C. According to the used temperature and silane concentration, two distinct types of particles can be obtained: at 625 °C, spherical particles with smooth surface and a low degree of aggregation, but at a higher temperature (650 °C) and lower silane concentration, particles with extremely rough surfaces and high degree of aggregation are found. This demonstrates the importance of the synthesis temperature on the morphology of silicon particles. The two types of silicon nanoparticles were subsequently used as active materials in a lithium half cell configuration, using LiPF_6_ in an alkylcarbonate-based electrolyte, in order to investigate the impact of the particles morphology on the cycling performances of silicon anode material. The difference in morphology of the particles resulted in different volume expansions, which impacts the solid electrolyte interface (SEI) formation and, as a consequence, the lifetime of the electrode. Half-cells fabricated from spherical particles demonstrated almost 70% capacity retention for over 300 cycles, while the cells made from the rough, aggregated particles showed a sharp decrease in capacity after the 20^th^ cycle. The cycling results underline the importance of Si particle engineering and its influence on the lifetime of Si-based materials.

## Abstract 2

To demonstrate the influence of the origin of Si materials on their performance in Li-ion batteries, Si nanoparticles were synthesized *via* silane pyrolysis. We highlight the importance of morphology engineering for creating long-lasting materials for Li-ion batteries.

## Graphical abstract

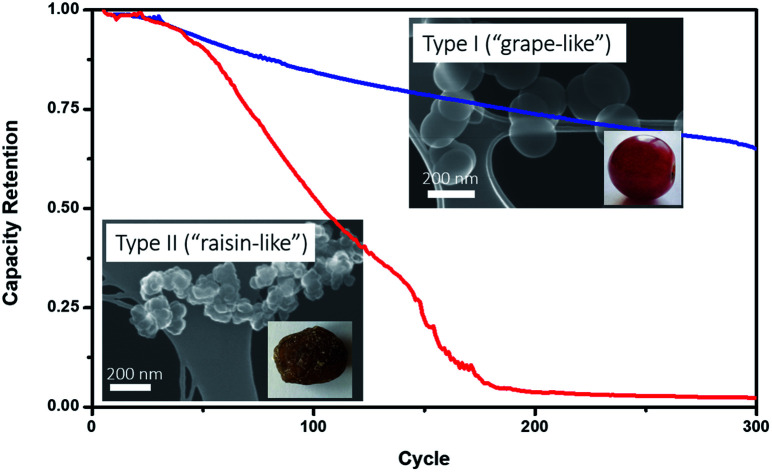

## 1. Introduction

The discovery and wide deployment of Li-ion batteries (LIBs) offered a suitable solution for the modern portable electronics, delivering high gravimetric and volumetric capacities.^1^ However, despite their prospects, present-day LIBs are not able to deliver the performance required to meet future demands. Therefore, significant research challenges and opportunities still exist in the design and synthesis of new materials which can deliver high energy densities and long cycle life for the next generation of LIBs.

Currently, the most frequently used anode material is graphite which offers the storage of one lithium ion per six atoms of carbon, providing a theoretical capacity of 372 mA h g^−1^. A number of alternative materials have been suggested to improve the anode capacity while enabling fast lithiation/delithiation rates.^2^ Silicon (Si) is one of the most popular choices among advanced anode materials due to its theoretical capacity of 3579 mA h g^−1^ by being able to alloy 3.75 lithium ions per silicon atom at room temperature.^3^ Si also delivers a low discharge potential, and therefore, high energy density batteries can be made if Si-based anodes are combined with an advanced high voltage cathode.^4^ However, these attractive properties also have drawbacks: unlike intercalation active materials, the alloying mechanism of lithium intake by Si leads to an enormous volume expansion (>300%).^5^ Such structural changes result in bulk Si cracking, leading to the exposure of new Si surfaces during lithiation and, thus, resulting in a constant formation of the solid electrolyte interphase (SEI) layer.^6^ Moreover, anisotropic lithium diffusion causes crack initiation perpendicular to the electrode surface, followed by SEI expansion through the electrode.^7^

Several approaches have been proposed to overcome the failure mechanisms outlined above not only from the perspective of materials development but also through optimization of other components.^8^ The preparation of porous Si or even Si hollow nanospheres has been suggested to counteract the expansion/contraction process.^9–13^ Nanostructuring could also be applied to Si to produce nanowires or thin Si layers which have been shown to have better stability compared to the bulk.^14–17^ Amorphous Si nanoparticles (Si-NPs) or Si nanocrystals (Si-NCs) can potentially provide a reasonable solution: utilizing a material with relatively small dimensions, typically a few hundred nanometres in size, can mitigate common degradation pathways while delivering an efficient solution-processing route similar to the one used in modern industry.^18^ The concepts of Si-NPs and Si-NCs have been widely explored over the recent years and has resulted not only in the demonstration of improved battery performance but also in the finding of a critical size for crystalline particles, which was estimated to be around 150 nm. This critical size refers to the largest Si-NCs which do not demonstrate bulk-like degradation behavior.^6^ Similarly, the critical size of amorphous particles was also estimated to be above 870 nm using *in situ* TEM.^19^ Importantly, it has also been proven that Li intercalation results in the destruction of the crystalline structure of Si, leading to a completely amorphous structure even after the first cycle.^20^

A variety of pathways for the synthesis of Si-NPs and Si-NCs have been proposed during the past decade. Despite the variability of silicon sources and synthetic methods, currently there is no clear evidence which route can potentially provide the “best” Si-NPs or Si-NCs in terms of their performance in batteries.^21^ The methods for synthesis of Si-NCs are represented by the colloidal approach which generally leads to small nanoparticles in the range of tens of nanometers.^22^ Other pathways include etching of Si,^23^ ball-milling,^24^ reduction of silicon dioxide,^25^ and the metallurgical route.^26^ While the majority of methods are focused on the preparation of crystalline silicon, the pyrolysis of silane gas was proposed as a suitable method for the preparation of amorphous Si-NPs.^27,28^ As evidenced by selection of this route by Si-production companies,^29^ silane pyrolysis provides a scalable, versatile route for the preparation of not only pure Si-NPs but also delivers an opportunity for the preparation of Si-based nanoparticles with various chemical compositions.^14^ The control of the gas flow, silane/hydrogen/argon ratio, and the temperature of the pyrolysis provides a set of tools to control the particle size and, possibly, the morphology of particles. Herein, we investigate the influence of the morphology of Si-NPs prepared by silane pyrolysis on the performance and lifetime of LIBs. In addition, the aim of the present work is to highlight the importance of material's history and material's selection for the reliable preparation of Si-based anodes.

## 2. Experimental

### 2.1 General

All manipulations with the Si particles were performed under ambient conditions. Silane and other gases (ultra-high purity) were purchased from Praxair. Special precautions should be in place while handing silane gas as it is pyrophoric. Unless specified, all chemicals used for the battery fabrications were purchased from Sigma-Aldrich and used without further purifications. The SEM analysis was performed using Hitachi S-4800 instrument operated at 30 kV in a transmission mode (STEM-in-SEM). Brunauer–Emmett–Teller (BET) surface area analysis was performed by a Micromeritics 3Flex. ICP analysis was carried out by Thermo Scientific Element XR High Resolution ICP-MS; hydrogen desorption analysis was performed by a Thermo Scientific Delta V IRMS (isotope ratio mass spectrometer) coupled to a Thermo Scientific FlashEA Elemental Analyzer. EIS was performed on Li half cells with electrodes of type I and II particles at OCV using a 10 mV amplitude and a frequency range from 10 mHz to 1 MHz. EIS was performed on 2032 coin cells using a BioLogic SP-300 instrument.

### 2.2 Synthesis of silicon nanoparticles

In the present work, Si-NPs were prepared by pyrolysis of silane gas (SiH_4_) in a free space reactor (FSR schematically shown on the Fig. S1 of ESI†) at temperatures ranging from 575 to 675 °C as described in an earlier work.^27^ The obtained particles are collected upon completion of the pyrolysis under ambient conditions, examined by low-voltage scanning transmission electron microscopy in a scanning electron microscope and stored for future use. The additional experimental details for the preparation of each batch used in the present work and additional characterization are shown in the ESI.†

### 2.3 Battery fabrication and electrochemical testing

The procedure was adopted from earlier work and modified to accommodate the amounts used in the present work.^30^ The slurries were prepared using the following general procedure: by weight, 15% sodium carboxymethyl cellulose (Aldrich) as binder, 10% graphite (TIMCAL KS6 L), 15% carbon black (TIMCAL Super C65), and the balance of silicon active material. The binder was first dissolved in 0.3 mL of a buffer solution with a pH of 2.87, made from KOH and citric acid, and centrifugally mixed (ARE-250CE, Thinky Co.) with 0.4 mL of deionized water (18.2 MΩ cm). Graphite, carbon black, silicon and an additional 0.3 mL of deionized water were added and mixed again. The slurries were then mixed using an ultrasonic mixer.

For the fabrication of electrodes, the slurry is gradually added in 150 mg batches and tape casted in repeated layers onto an 18 μm thick dendritic copper foil (SE-Cu58, Schlenk) with doctor blade thickness of 76.2 microns (3 milli-inch) using a Hohsen MC20 mini-coater. After casting and drying overnight, the electrodes were dried in a vacuum oven at 120 °C for 3 hours, punched into 15 mm discs for half-cells assembly and weighed. Active material loading varied from 0.16 mg cm^−2^ to 0.38 mg cm^−2^, depending on the slurry, however the variation was less than 0.10 mg cm^−2^ within a given slurry or set of electrodes. The average loading of type I electrodes was 0.20 mg cm^−2^ and that of the type II electrode was 0.30 mg cm^−2^. Cells for a given Si-NPs batch were assembled in identical sets of three in an Ar-filled glovebox (MBraun) with less than 0.2 ppm of oxygen and water vapor. Coin cells were assembled using 2032 stainless steel casings with Li foil counter electrodes (99.99%, LinYi Gelon LIB Co., 15 mm in diameter and 0.250 mm thick) in a 2-electrode configuration. Monolayer porous polypropylene films (Celgard H2400) were punched into 18 mm discs for use as separators. The electrolyte composition was 1.2 M LiPF_6_ in 3 : 7 ethylene carbonate:ethylmethyl carbonate (EC : EMC), respectively, with 10 wt% of fluoroethylene carbonate (FEC) and 2 wt% of vinylene carbonate (VC) as additives, purchased from Solvionic. 35 μL of electrolyte was added using a micro-pipette to adequately wet the electrode and separator. The best performing data was chosen from each set. Half cells were tested using an Arbin BT-2000 cell tester between 0.05 V and 1.00 V *vs.* Li^+^/Li with three initial formation cycles at C/20 (0.18 A g_Si_^−1^) followed by continuous cycling at C/10 (0.36 A g_Si_^−1^). The cells for post-mortem analysis were delithiated and disassembled in the Ar-filled glove box, washed with dimethyl carbonate (DMC), dried, and examined by SEM.

## 3. Results and discussion

### 3.1 Characterization and morphology of silicon nanoparticles

In the present work Si-NPs were prepared by flowing silane gas (SiH_4_) in the presence of hydrogen through a hot-wall free space reactor (FSR) heated to temperatures ranging from 575 to 675 °C. Higher pyrolysis temperatures may result in higher nucleation rates leading to the formation of smaller particles. Similarly, nucleation and growth can be modulated by changing concentration of precursor which affect the size and morphology of the particles. If only the temperature is modified, it is likely that both the particle size and morphology would be altered simultaneously, thereby confounding comparative studies.^31^ Additionally, even within the same preparation route there are various factors which could influence the growth of Si particles.^32,33^ Herein, the combination of temperature and silane concentration for pyrolysis was chosen with the intent to achieve a specific size and morphology of particles while maintaining similar size distribution and collection efficiency between batches.

**Fig. 1:**
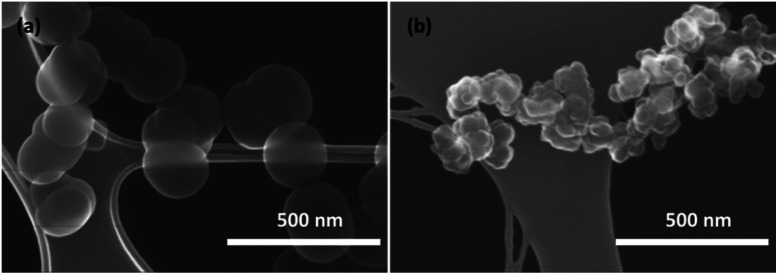
SEM images at low magnification of Si particles obtained at (a) 625 °C (type I) and (b) 650 °C (type II).

The modifications of the synthesis conditions resulted in noticeable changes in the Si particles' shape and size as illustrated by the SEM images displayed on Fig. 1, which reveals two distinct types of particles. Smooth spherical particles, as shown in Fig. 1a were obtained at lower temperature and denoted here as type I particles. Smaller particles with relatively irregular surfaces referred as type II are shown in Fig. 1b and were obtained at higher pyrolysis temperatures. Spherical particles of type I have sizes averaged at approximately 200 nm with smooth edges and a low degree of aggregation, while type II particles are smaller (around 50 nm in average) and characterized by rough surfaces and high aggregation (the size histograms area shown on Fig. S2 and S3†). The exact size of type II primary particles is difficult to estimate owing to their uneven shape and rough surface, resembling a popcorn- or raisin-like morphology. Further *HRTEM* analysis revealed that the type II particles consist of several sub-particles of an even smaller size (around 20 nm) fused together and representing a single particle after isolation (Fig. S4†). The formation of such particles is caused by rapid nucleation during synthesis due to high temperature during synthesis, resulting in the simultaneous formation of many particles which collide together and partially sinter during further growth. Despite the morphological differences between types I and II particles, both are composed of amorphous Si, which was confirmed by the absence of corresponding peaks in X-ray diffraction (Fig. S5†). The chemical purity of Si-NPs was additionally confirmed by ICP and hydrogen desorption analysis (ESI).† The latter was performed to demonstrate that both particles' types have small amounts of residual hydrogen embedded in the particles, which originates from the pyrolysis process.

Even though further exploring is needed to precisely determine the synthesis conditions/factors leading to either type I or type II particles, the primary mapping of the pyrolysis conditions has demonstrated that higher temperatures and lower silane concentrations lead to type II particles. To ensure the reproducibility, two batches of each type were synthesized (Table S1†). From all batches synthesized in the present work, we have observed either pure fractions of the type I or II or, occasionally, fractions with a mixture of both types (Fig. S6†). No intermediate morphologies demonstrating a gradual transition from type I to type II have been evidenced as could be seen from the low-resolution SEM images shown in Figs. S7 and S8 of ESI.† Furthermore, while some size control could be achieved for the type I through the change of the reaction conditions, the size and morphology of type II was not sensitive to variations in the pyrolysis conditions within the limits of the present method.

### 3.2 Electrochemical performance of silicon nanoparticles

**Fig. 2:**
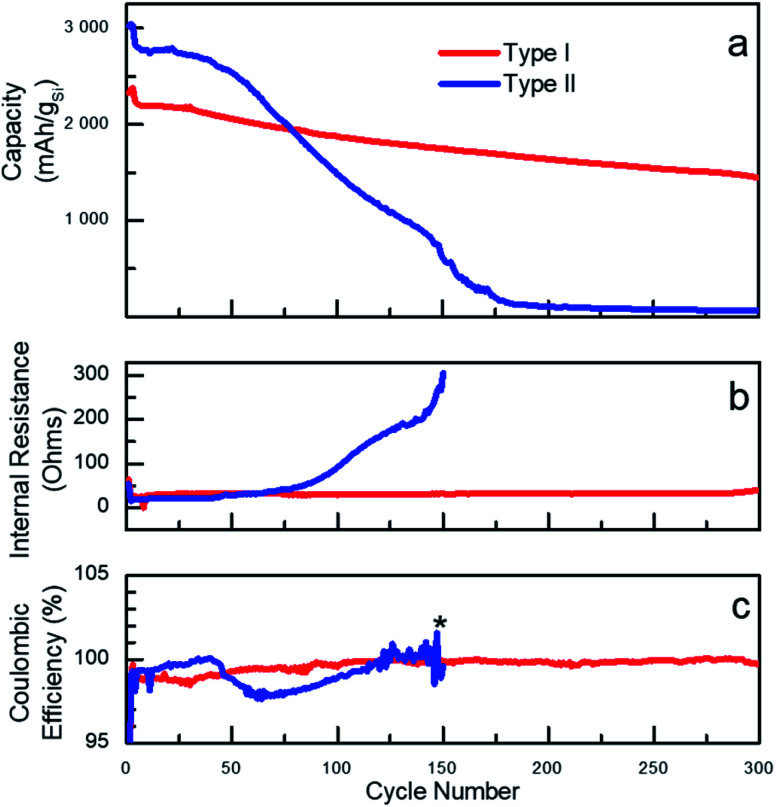
(a) Comparison of specific lithiation/delithiation capacity for type I and type II particles in half-cell configuration using LiPF_6_ in EC/EMC with VC and FEC additives as electrolyte; (b) internal resistance; (c) coulombic efficiency of batteries fabricated from type I and type II particles. (*) Internal resistance and coulombic efficiency for type II is only shown for the first 150 cycles (further measurements were not relevant past this point due to the cells failure).

The vast majority of the work assessing performance of Si-based materials in LIBs has been performed on crystalline materials.^20^ However, based on known degradation processes,^19^ it is anticipated that the shape of amorphous Si-NPs prepared in the present work will also have a significant effect on performances of Si-based battery anodes. To illustrate such effect, sets of batteries utilizing type I and type II nanoparticles were fabricated in half-cell configuration with a Li foil as the counter electrode. For cycling the fabricated cells, galvanostatic conditions were applied at room temperature and the corresponding electrochemical performance is shown in Fig. 2. The specific delithiation capacity of the silicon particles of type I and II is plotted on Fig. 2a against the cycle number. To specifically emphasize the dependence of the performance on the particle type, two batches of Si-NPs of types I and II were utilized for battery fabrication, both demonstrating almost identical behaviour for the same particle type (Fig. S9†).

A common distinguishable feature of most of Si-based electrodes is high initial lithiation/delithiation capacity,^7^ which was also observed for both Si-NP types studied in the present work. However, the initial capacity values were observed to be only slightly higher for the electrodes prepared from type II particles. Cell cycling began with three formation cycles, which are necessary for the initial uniform lithiation of the Si-NPs and are typically performed at low current density (C/20, 0.18 A g_Si_^−1^). The first slow lithiation cycles minimize the initial structural damage caused by lithium penetration into pristine Si-NPs, thus extending the lifetime of the active material. Upon completion, the electrode capacity usually drops over the next few cycles due to the increase in current density to C/10 (0.36 A g_Si_^−1^). Such behaviour is common for Si-based electrodes and was also observed for both type of particles studied herein, demonstrating the independence of those processes on the particle morphology.^34^ The plot on Fig. 2a clearly illustrates a strong dependence of the battery performance on the nanoparticle's morphology/shape. Both types of particles visibly demonstrate non-negligible performance decay after the first cycles, but the electrodes prepared using type II nanoparticles degrade remarkably faster. Specifically, electrodes made from type II nanoparticles had higher initial capacities but demonstrated fast degradation starting after approximately 20 cycles as previously reported.^35^ However, the electrodes prepared from type I particles notably retained almost 70% of the capacity over 300 cycles (formation cycles are excluded).

The degradation can be interpreted through different capacity fading rates as visualized on the capacity plots. For instance, for electrodes prepared from type II Si-NPs, it is possible to distinguish two stages of degradation – before cycle 50 (slow) and after (fast). Such differences point to the presence of several mechanisms of cell degradation. We speculate that the initial decay of the cell capacity is due to initial microscopic changes in the particle's shapes and SEI formation. All these changes result in a drop of the coulombic efficiency (CE) around cycle 50 for type II particles.^36^ (CE is defined as ratio of the delithiation charge to lithiation charge). The degradation of the electrodes prepared from the type II particles not only result in a drop of capacity but also in a slow increase of the internal resistance (IR, Fig. 2b). This is likely caused by continuous SEI formation, exacerbated by the large surface area and the rough surface of type II particles. The type II electrode has lower initial IR than the type I electrode, which was confirmed by EIS (Fig. S10†). After completion of approximately 50 cycles, we believe that the degradation becomes macroscopic owing to excessive SEI formation which eventually fills up the pores in the electrode structure, thus compromising the long-range ionic conductivity throughout the electrode as well as contributing to the disruption of the electrical conductivity. A large and sudden increase in the internal resistance along with fast capacity decay serves as an indirect indication of these processes. By the 150^th^ cycle, the cells made of type II particles failed completely.

Initially, the difference in electrochemical performance was hypothesized to be owed to the difference in average particle size between type I and type II. To validate this explanation, two batches of type I Si-NPs with sizes equal respectively to 80 nm and 200 nm, were prepared and electrochemically tested within the present work (Table S1 and Fig. S9†). However, only a minor difference in electrochemical behaviour was observed for these two batches, which could be attributed to variation in electrode preparation and cell assembly. Hence, the degradation of electrodes made from type I Si-NPs does not change as a function of the particle size within the studied range, which indirectly supports earlier estimations of the critical sizes for amorphous Si performed by TEM.^19^ The same could be extended to the type II Si-NPs and therefore, it is reasonable to propose that for sizes below the critical size the surface morphology plays a more important role than the particle size.

**Fig. 3:**
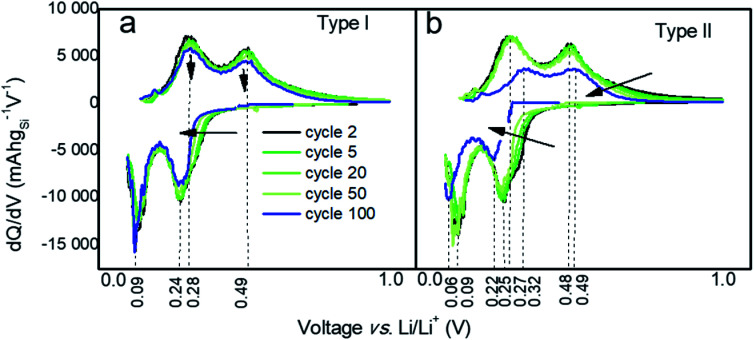
Differential capacity curves at cycles # 2, 5, 20, 50, and 100 for electrodes prepared from (a) type I and (b) type II Si-NPs.

**Fig. 4:**
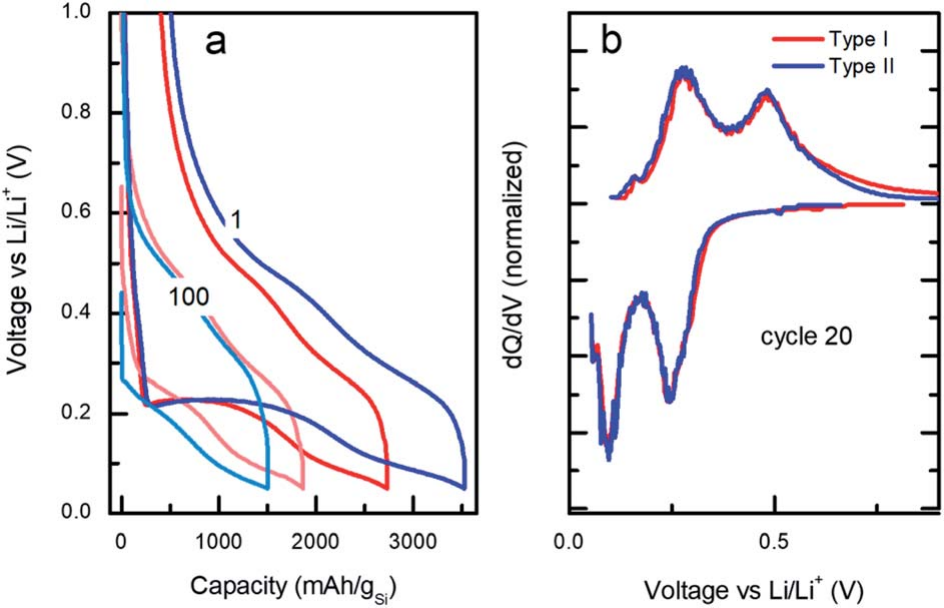
(a) Voltage/capacity galvanostatic curves at cycles 1 and 100 and (b) differential capacity *vs.* applied voltage profiles at cycle 20 for type I (red) *vs.* type II (blue).

To gain further understanding into the differences in performances of the two types of particles, differential capacities (d*Q*/d*V*) were plotted against the voltage as shown in Fig. 3 and 4. Both types of Si-NPs exhibit two primary lithiation and delithiation peaks as commonly observed for Si-based anodes (Fig. 3).^37^ The positions of the peaks shift over the course of testing, indicating an increase in internal resistance within the cell. Such shifts are more pronounced for the electrodes made of type II particles where both lithiation and delithiation processes possibly become slowed. In addition, the more pronounced capacity fading for type II particles is also seen in this plot by the diminishing area under the curves, which is linked to the lithiation and delithiation capacities.

Despite the large differences in capacity fade for the two types of particles, the voltage/capacity and d*Q*/d*V* curves displayed on the Fig. 4a and b, respectively, demonstrate similar lithiation/delithiation mechanisms for both types of particles as expressed by the two lithiation plateaus commonly seen for Si-based electrodes.^38,39^ However, comparison of the voltage/capacity curves at the first cycle for the different particle types indicates higher irreversible capacity of the electrodes prepared from the type II particles. This pattern continues through the initial cycling, suggesting higher SEI formation for the electrodes prepared from type II particles (Fig. S11 and S12†) and evidenced by the growth of the cumulative irreversible capacity (Fig. S13†). As will be discussed later, the build-up of the SEI is one of the most likely causes of the cell's ultimate degradation and failure.

### 3.3 Degradation analysis

The microscopic mechanism of degradation suggested above should manifest itself at the early stages of the battery life. Nevertheless, a closer analysis of the normalized differential capacities at cycle 20 in Fig. 4b shows a tailing of the delithiation current for the type I particles at the highest voltages, which may be an indication of slower diffusion process for lithium in type I Si-NPs at that stage of the battery life, due to the larger diffusion distances in these particles.

**Fig. 5:**
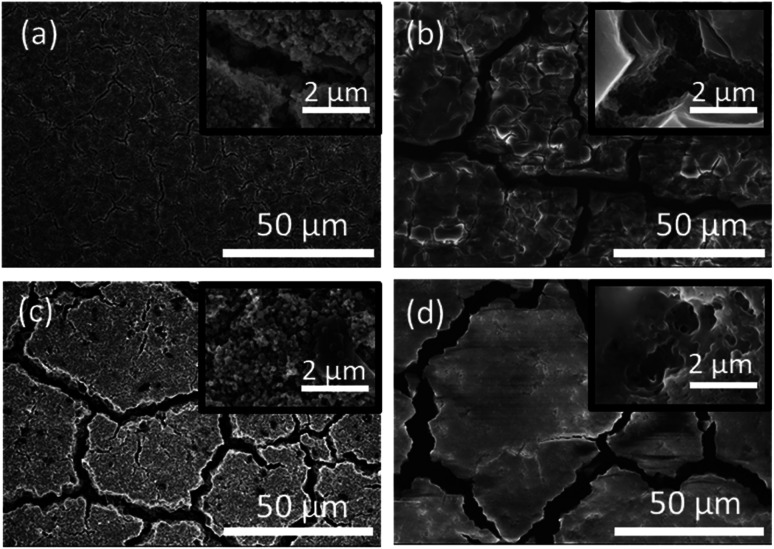
Post-mortem SEM imaging of electrodes fabricated from type I Si-NPs (a and b) and type II Si-NPs (c and d) after 3 formation cycles performed at C/20 (a and c) and after 300 additional cycles performed at C/10 (b and d). The insets show the corresponding high-resolution images.

Post-mortem SEM was employed after cell cycling to evaluate how the nanoparticle morphology influenced the electrode stability after the first three formation cycles and towards the end of cell's life. Half cells were cycled 3 times at C/20 and paused for 24 hours after the final delithiation, disassembled, washed with DMC, and mounted for SEM analysis. The same process was applied to the cells which had been cycled 300 times at C/10 rate. Fig. 5 contains SEM images for both Si-NPs types at two different stages of the battery life. It is apparent that after the first 3 cycles the Si-NPs still largely retain their initial morphology as indicated by how individual particles are resolvable. The particles of both types are all coated with SEI and no detectable changes due to initial cycling are observed. The low magnification SEM images are shown in Fig. 5. On the macro scale (50 μm), even at the early stages of the electrode's cycle life, crack formation was observed. It is noteworthy that the electrodes made of type II particles experienced larger and more pronounced cracks than those made of type I particles. After 300 cycles, the cracks continue to develop in size; however, their pattern is predetermined after the initial cycling (Fig. 5b and d). Within the electrode domains separated by cracks, it can be seen in Fig. 5d that type II electrodes exhibited a smoother, denser, and more filled-in morphology compared to the smaller fissures in Fig. 5b that are still visible in the interior of type I electrode domains after 300 cycles. High-resolution images further indicate the difference morphology at the primary particle level. For comparison, SEM images of the electrodes prior to cycling are shown in Fig. S14.† It is apparent that the pristine electrodes did not shown any cracks or defects which were observed after even initial cycling.

Based on the initial particle's morphology observed in SEM, the specific surface area was expected to be higher in type II particles. BET measurements of type I and type II specific surface areas were 31 m^2^ g^−1^ and 35 m^2^ g^−1^, respectively (full isotherms plotted in Fig. S15†). Due to their higher specific surface area and higher apparent specific capacity as demonstrated earlier, type II particles expand more than type I during the initial lithiation cycle, during which the SEI layer is formed. Thus, the relative volume changes for type II particles will be larger leading to more pronounced macroscopic changes. In addition, the nanostructured surfaces may result in a higher migration of silicon atoms and structural disintegration during lithiation/delithiation sequences, when parts of the particles delaminate and lose electrical connectivity. Additionally, since the formation of type II particles arises from the aggregation of smaller nanoparticles, the surface oxide layer may not form into a well-defined layer, which can give rise to a poor cyclability.^40^ These factors are all contributing to the fast “macroscopic” cell degradation mentioned earlier.

In contrast, the slightly lower capacity of type I Si-NPs leads to less lithium intake, inducing smaller volumetric changes. The smooth morphology of the particles and the resulting uniform expansion/contraction enable better control of the build-up of the SEI, as indirectly confirmed by the electrochemical evaluation discussed above. In essence, the morphology of type I Si-NPs confers a similar benefit as “limited capacity” cycling, which is well known to extend overall cell cycle life. The specific surface area is lower than type II, but at the 1^st^ cycle, the CE was approximately the same as that of type II nanoparticles. Thus, the structural influences of particle's morphology on SEI take several cycles to propagate into macroscopic effects compared to the immediate effects on the CE caused by large differences in particle size.^41^ By comparison of the SEM images displayed in Fig. 5(b) and (d), after 300 cycles, it is obvious that the electrodes prepared from type II particles are less porous than those prepared from type I particles. Notably, the crack pattern does not significantly change even after 300 cycles and the initial electrode structure is preserved.

**Fig. 6:**
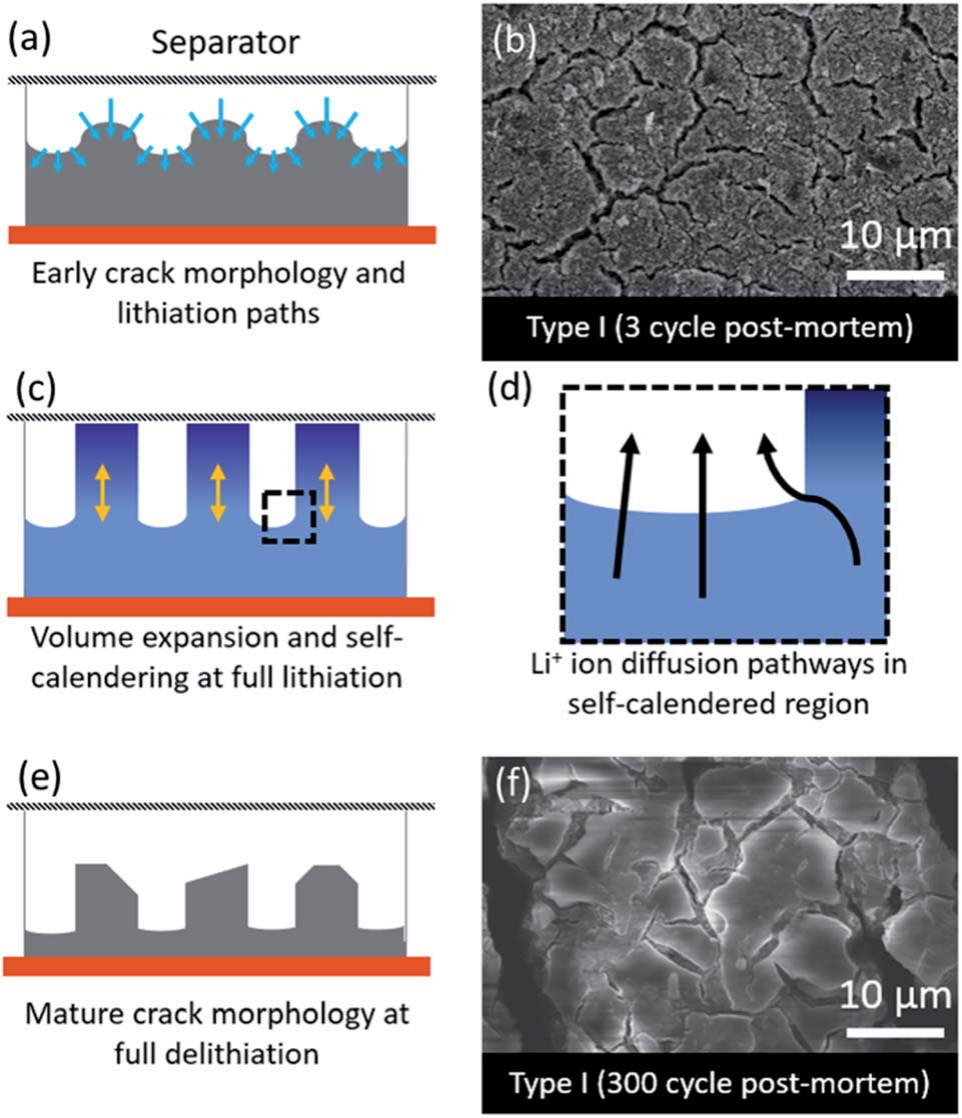
Schematic illustration of self-calendaring effect based on post-mortem analysis of the anodes: (a) proposed initial fracturing of the silicon-based electrode during lithiation; (b) post-mortem SEM images of delithiated electrodes composed of type I particles after 3 lithiation/delithiation cycles; (c) proposed morphological changes for the fractured lithiatted Si-based electrodes; (d) Li ions diffusion paths for fractured electrodes; (e) proposed morphology after delitiation of a mature electrode; (f) post-mortem SEM images of deliuthiated electrodes composed of type I particles after 300 lithiation/delithiation cycles.

The fast degradation of the electrodes prepared from type II Si-NPs can potentially be further rationalized through a hypothesized “self-calendering” phenomenon as schematically illustrated in Fig. 6. Within the confined space of a rigid coin cell, the effect of volume expansion and continuous SEI growth leads to compaction of the initially porous electrode, a process which depends on the particle morphology. The formation of initial fractures in the electrode dictates further lithiation and delithiation pathways as well as templating the final electrode morphology. The denser sections inside the electrode subsequently delithiate more slowly while active material nearby the macroscopic cracks is more accessible to the electrolyte, leading to faster delithiation and associated volume contraction in that region. After 300 cycles, the result is an island-like morphology with deepening crack growth (Fig. 6a, c, and d). Due to continuous growth of the SEI layer accompanied with the expansion/contraction of the active material, the electrode expands vertically. This vertical expansion results in the electrode pressing against the separator and the rigid counter electrode and hence compacting the electrode (Fig. 6c). A post-mortem SEM comparison of type I and type II electrode morphologies (Fig. 5) reveals the severity of the clogging and loss of porosity. The self-calendering phenomenon leads to clogging of the electrode inside the forming islands, blocking diffusion pathways, disrupting electrical contacts, and hence causing loss of capacity despite the fact that Si active material may still be available. The loss of the electrical contacts due to increased resistance at the interfaces has also been identified as a potential degradation pathway in earlier studies.^42^ Further post-mortem studies, or *in situ* electron microscopy studies, will be necessary to confirm the occurrence of the self-calendering phenomenon for electrodes expanding in confined spaces against rigid boundaries.

## 4. Conclusions

Spherical Si nanoparticles with a smooth surface have been synthesised by means of silane gas decomposition in a free space reactor. The importance of the synthetic conditions has been underlined for the preparation of Si-NPs and, hence we demonstrate the importance of engineering the morphology and not only the size of Si-NPs. As expected, the electrodes fabricated from type I particles exhibit improved performance in half cells as compared to what is typically obtained from *pristine* Si particles of similar size. Upon cycling, the degradation of the Si-based anodes depends not only on the size of the particles, as was indicated earlier,^6,43^ but also on the shape of Si-NPs as morphological differences result in two distinct rates of electrode degradations linked to SEI formation and ultimately to a different battery lifetime. By using pristine type I Si-NPs, almost 70% of the initial capacity is retained after 300 cycles with a negligible contribution of the particle size in the range investigated. Owing to their smoother surface and better homogeneity, the cycling behaviour of type I Si-NPs is largely superior to type II Si-NPs. Thus, it is reasonable to conclude that the engineering of the initial particle morphology allows one to control the volumetric changes during lithiation/delithiation of the anode and such differences in expansion dictate further degradation of the electrode and the cell as a whole. In conclusion, it is important to emphasize that making comparisons of performance across different types of Si materials strongly depends on the material's history, preparation pathway, and morphology. For amorphous Si-NPs, size and morphology are the key factors that can mask the causes of performance degradation or enhancement, particularly when Si-NP architectures become more complex with coatings, doping, or core–shell modifications.

## Conflicts of interest

There are no conflicts to declare.

## Supplementary Material

NA-002-D0NA00770F-s001
