# Supplementary material for: Morphology engineering of silicon nanoparticles for better performance in Li-ion battery anodes
Source: Nanoscale Adv. 2020 Oct 13;2(11):5335–42. doi: 10.1039/d0na00770f (PMC9417716; doi:10.1039/d0na00770f)
Supplement: NA-002-D0NA00770F-s001 [file NA-002-D0NA00770F-s001.pdf]

This journal is © The Royal Society of Chemistry 2020

The free space reactor (FSR) for the preparation of the Si NPs was constructed using 50-mm diameter stainless steel tube with multiple heating zones, allowing gradual heating of the silane gas needed for undisturbed flow. Upon passing the heating zone, the formed particles are driven into the cold zone kept at 10°C to quench the pyrolysis process. During the preparation of multiple batches, each fraction was driven to a separate filter through the system of valves controlling the gas flow, from which the particles could be collected upon completion of the pyrolysis. After preparation of each batch, the FSR was flushed with argon to minimize potential cross-contamination between the batches. The formation of the particles was verified through an optical window embedded in the reactor after the quenching zone; particles were illuminated with a broadband light source and recorded with a camera to remotely visually observe particle formation. Upon completion of the pyrolysis process, the FSR was flushed with argon to ensure the complete removal of the silane gas, then the system could be opened, and the particles could be collected. The experimental conditions used for the preparation of each batch used in the present work are listed in Table 1. Concentrations of silane were varied from 17 to 25% by volume with the balance of hydrogen. The forming particles were vacuum filtered from the gas flow continuously until the filter is nearly clogged, as indicated by the progressive pressure drop measured downstream of the filter. Each batch is made within the same reactor by isolating the particles into individual filters driven by individually assigned valves. It should be noted that the relations between temperature, silane concentration, and particle size described in the present work is reactor dependent. However, the general trends should not depend on a particular setup given the similar architecture of the reactor. It should be noted that FSR used in the present work is tuned to produce relatively large volumes of the Si nanoparticles (up to 10 grams), while FSRs tuned to produce minimal amounts may deliver better flexibility and tunability.

Table S1. Silane concentration in volume % and reactor temperature used to produce different batches of Si nanoparticles.

Sample number	SiH_4 concentration (%)	Temperature (°C)	Average particle sizes	Particle type
Batch 1	25	600	80 nm	I
Batch 2	20	625	200 nm	I
Batch 3	20	650	70 nm	II
Batch 4	17	660	50 nm	II

In addition to microscopy and XRD analysis the elemental composition of obtained particles was also analysed by using ICP analysis and hydrogen desorption analysis. Due to specifics of the synthetic methodology, the presence of other elements in the nanoparticles was not anticipated: that was confirmed by the ICP analysis for a Si sample prepared through silane pyrolysis, as shown below:

Element	***B***	***Pb***	***Al***	***P***	***Ti***	***V***	***Cr***	***Mn***	***Fe***	***Co***	***Ni***	***Cu***	***Zn***
Conc (ppm)	*< 31.3*	*< 0.6*	*186*	*42*	*< 3.4*	*< 1.6*	*< 1.6*	*< 1.7*	*35.9*	*< 1.1*	*< 1.7*	*< 1.2*	*< 1.4*

The anticipated difference between the samples of type I and type II would be different amount of hydrogen, embedded in the nanoparticles. The hydrogen desorption analysis revealed no detectable difference between type I and type II samples: 0.036 wt % of hydrogen was detected for type I, and 0.037 wt % of hydrogen was detected for type II.

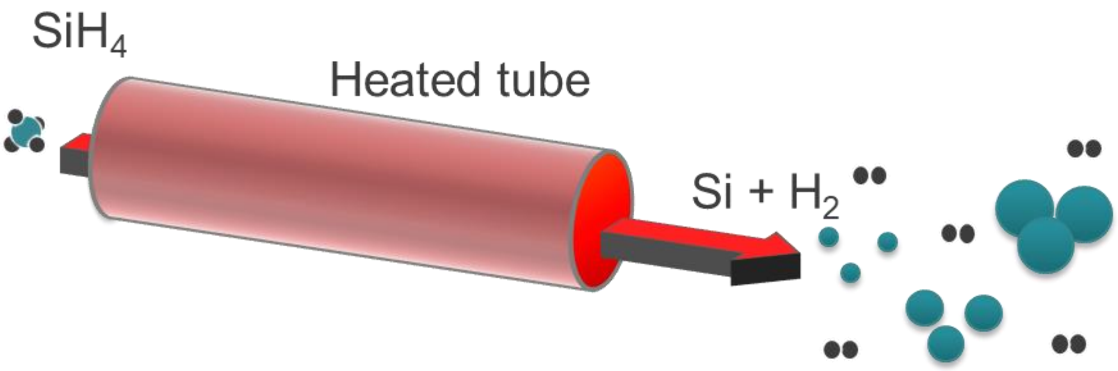

Figure S1. Schematic representation of FSR; collecting, heating and gas suppling infrastructure is omitted for clarity.

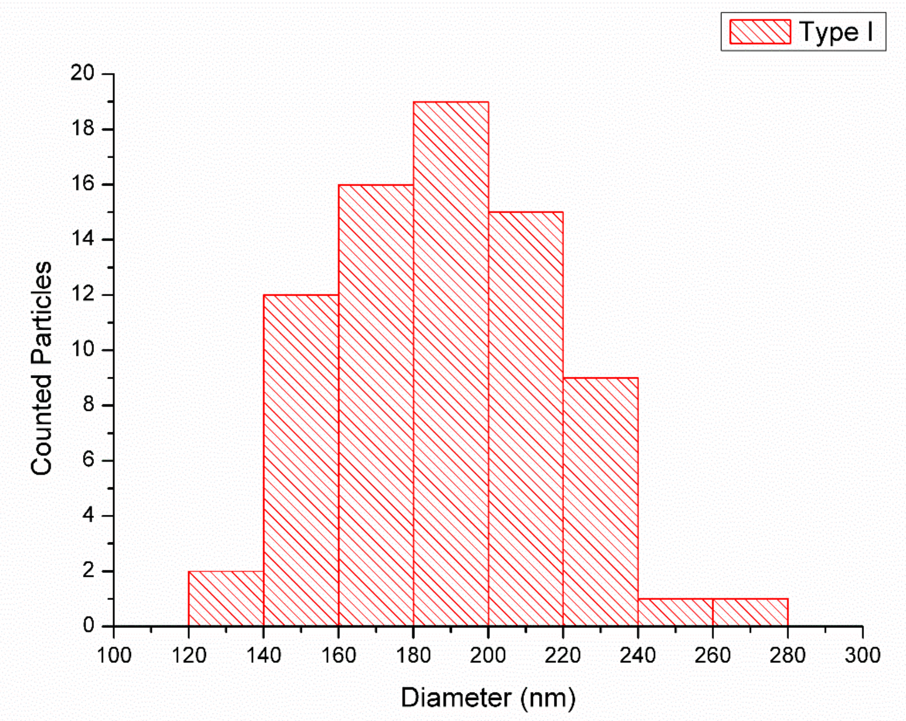

Figure S2. Histogram demonstrating the size distribution for type I Si nanoparticles.

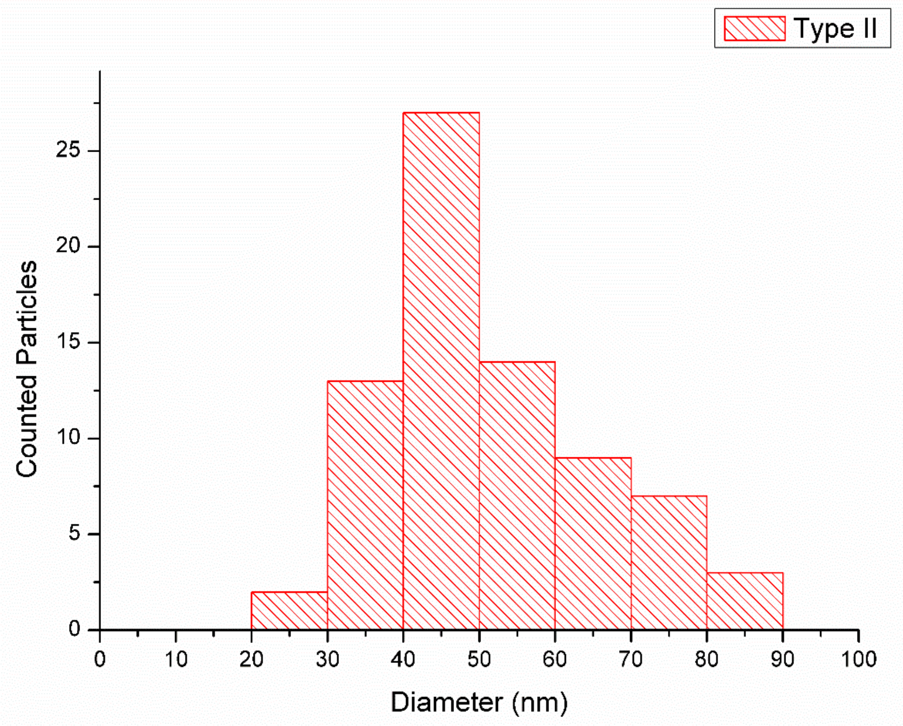

Figure S3. Histogram demonstrating the size distribution for type II Si nanoparticles.

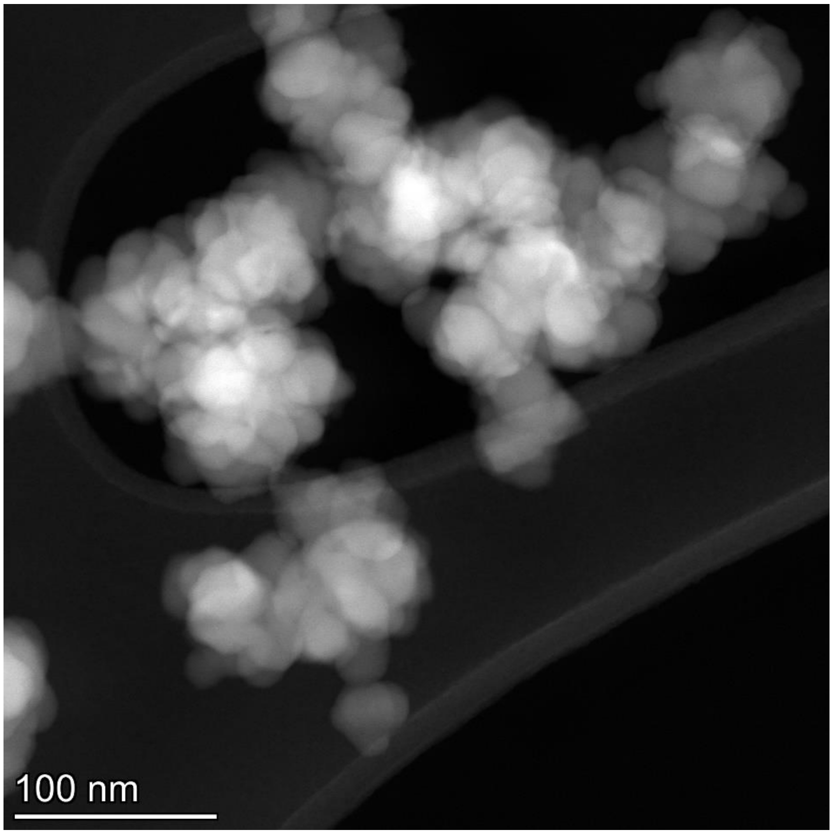

Figure S4. STEM HAADF imaging of type II Si nanoparticles.

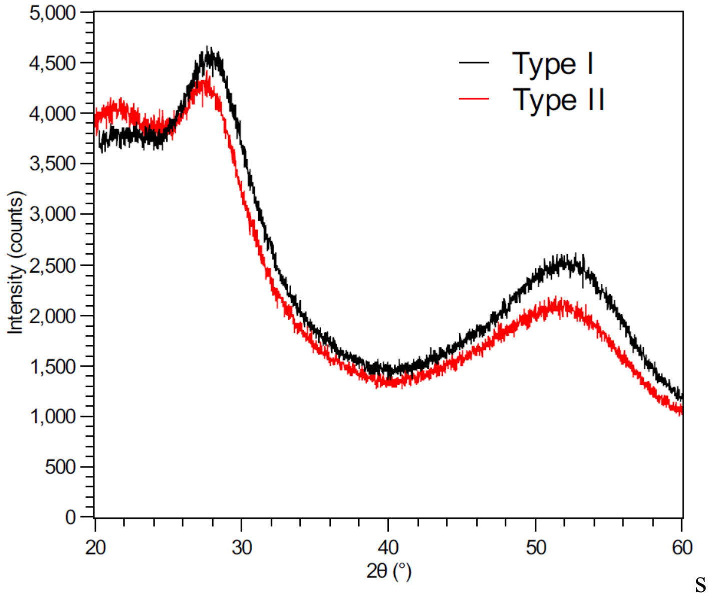

Figure S5. XRD pattern for Si nanoparticles confirming the amorphous structure.

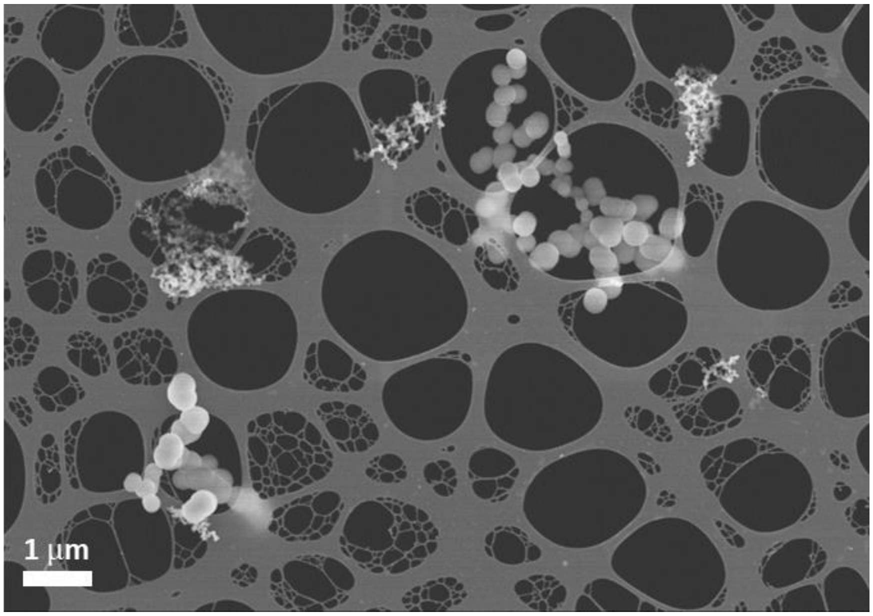

Figure S6. Low resolution SEM for the fraction containing a mixture of type I and II Si nanoparticles.

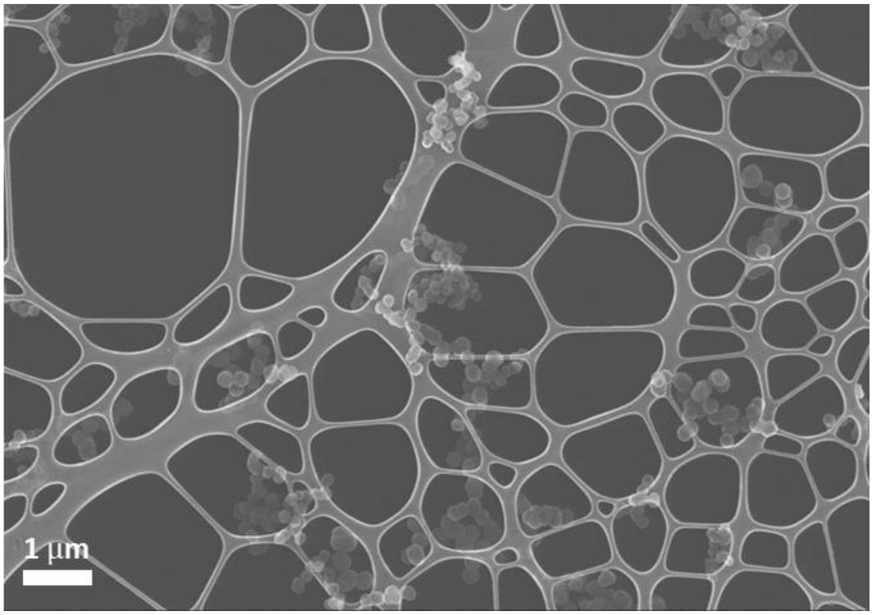

Figure S7. Low resolution SEM for type I Si nanoparticles.

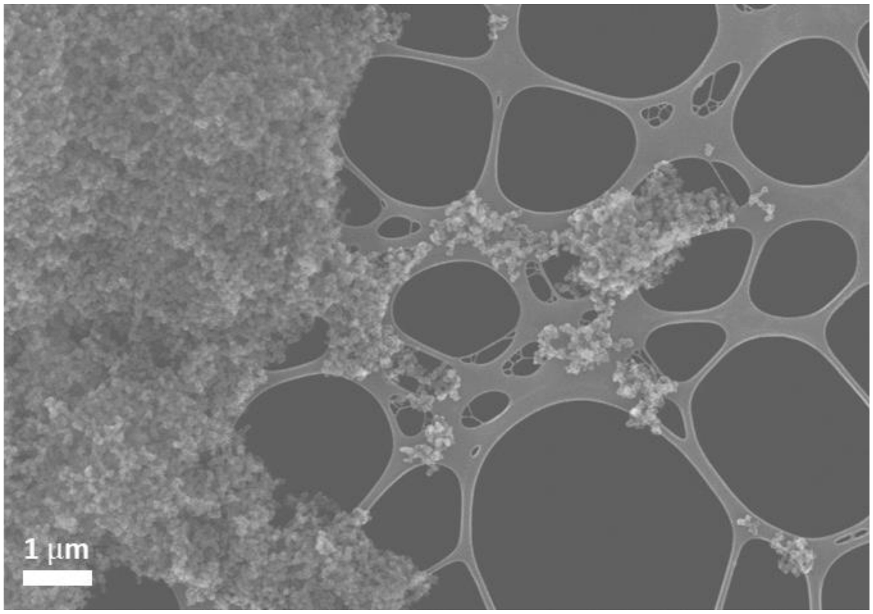

Figure S8. Low resolution SEM for type II Si nanoparticles.

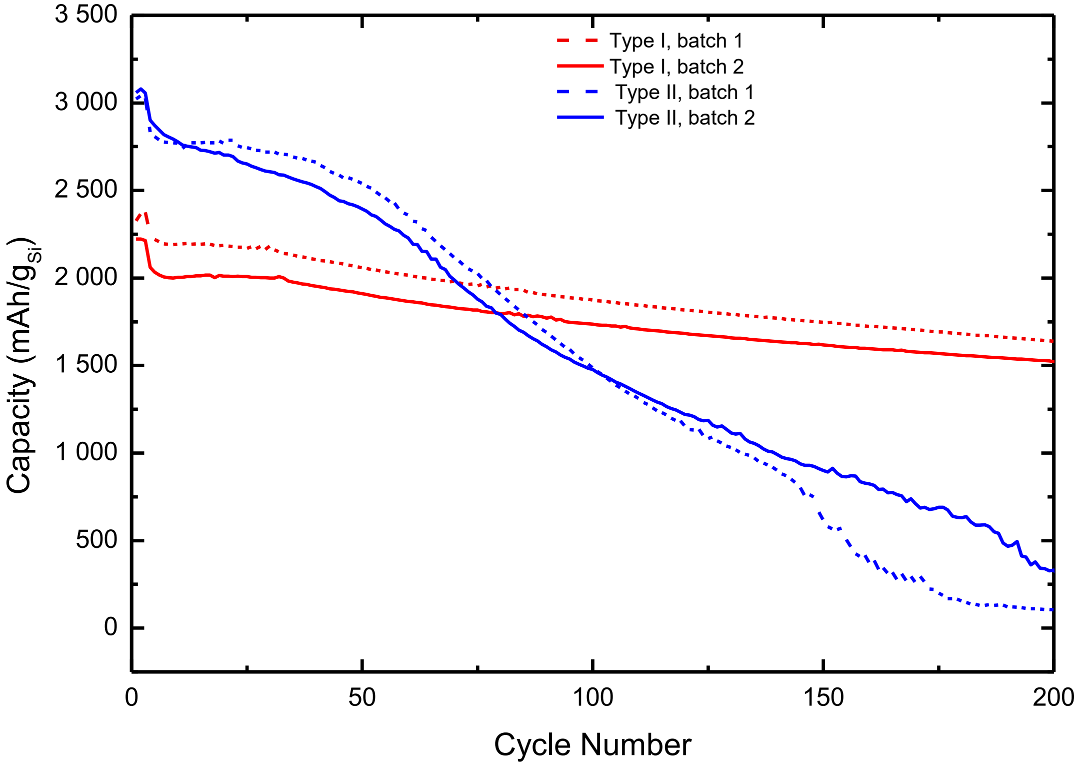

Figure S9. Comparison of specific charge/delithiation capacity of several batches of type I and type II Si-NPs in half cell configuration using a $LiPF_6$ in EC/EMC electrolyte-based system.

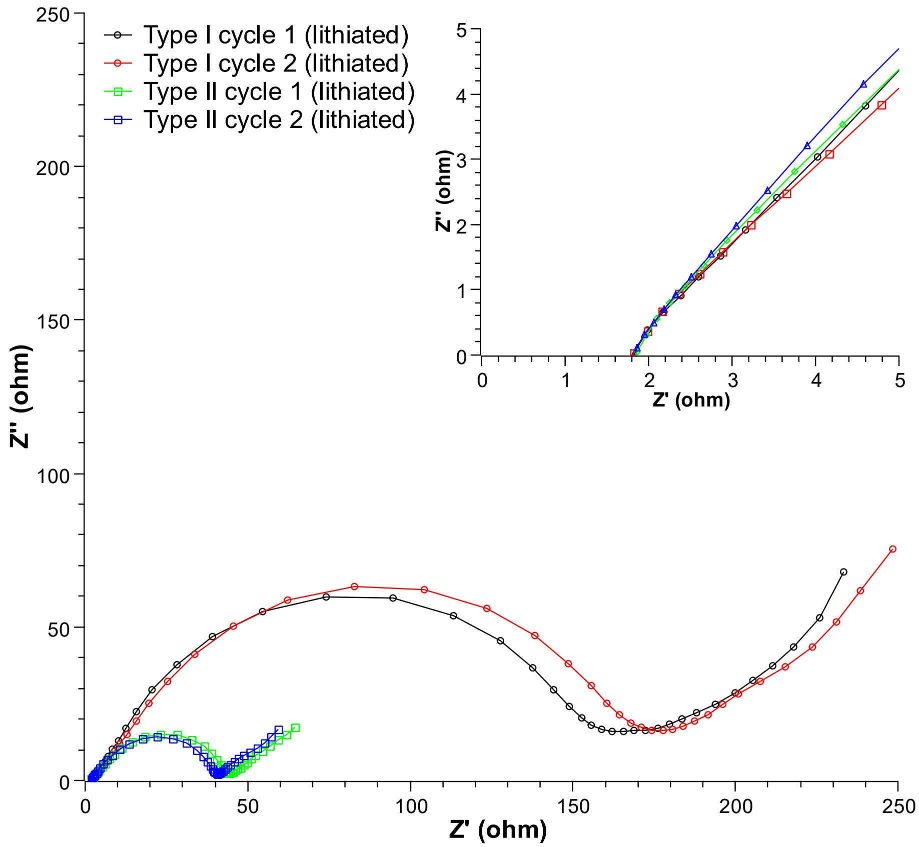

Figure S10. Comparison of electrochemical impedance spectroscopy (EIS) after cycle 1 and 2 of lithiated electrodes containing type I and type II Si-NPs in half cell configuration using a $LiPF_6$ in EC/EMC electrolyte-based system.

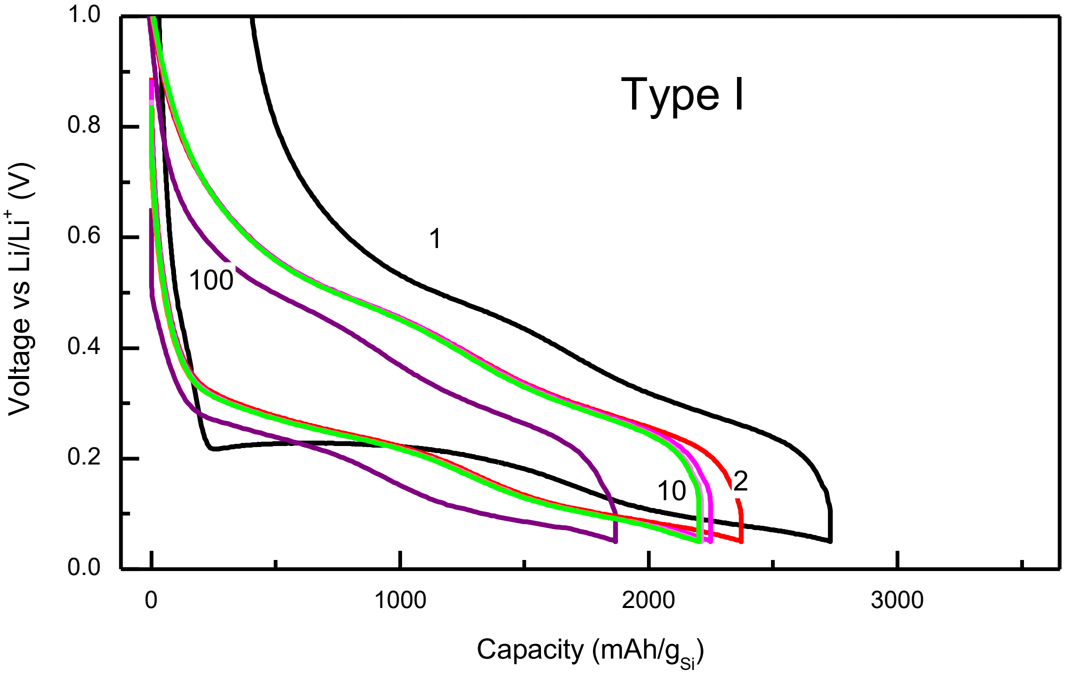

Figure S11. Voltage/capacity galvanostatic curves at cycles 1, 2, 4, 6, 8, 10 and 100 for the electrodes prepared from type I particles.

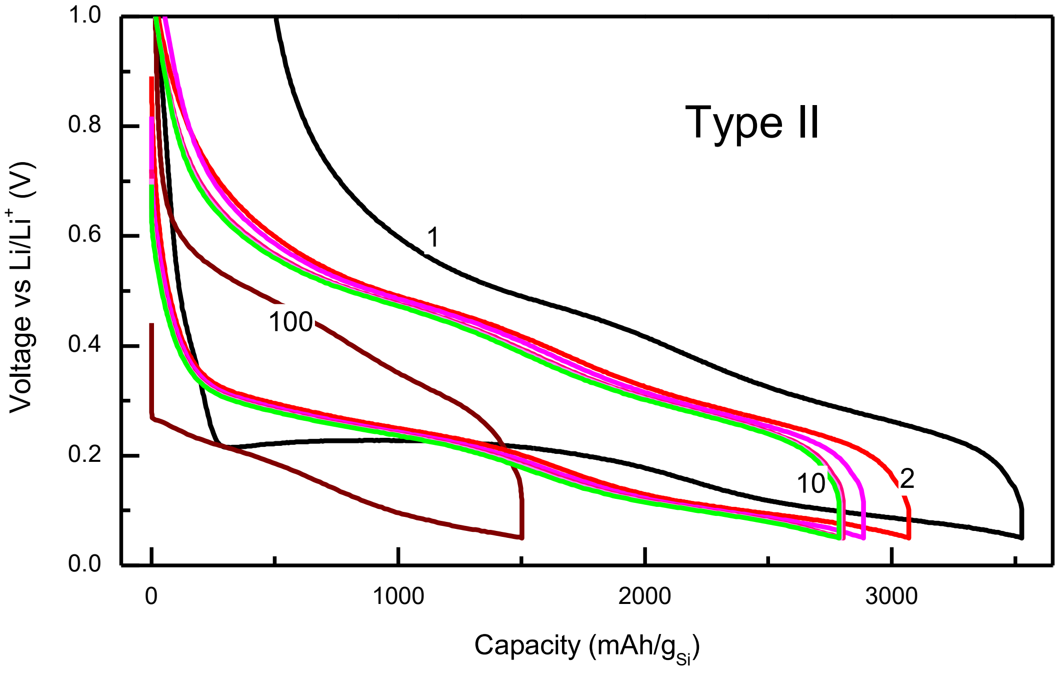

Figure S12. Voltage/capacity galvanostatic curves at cycles 1, 2, 4, 6, 8, 10 and 100 for the electrodes prepared from type I particles.

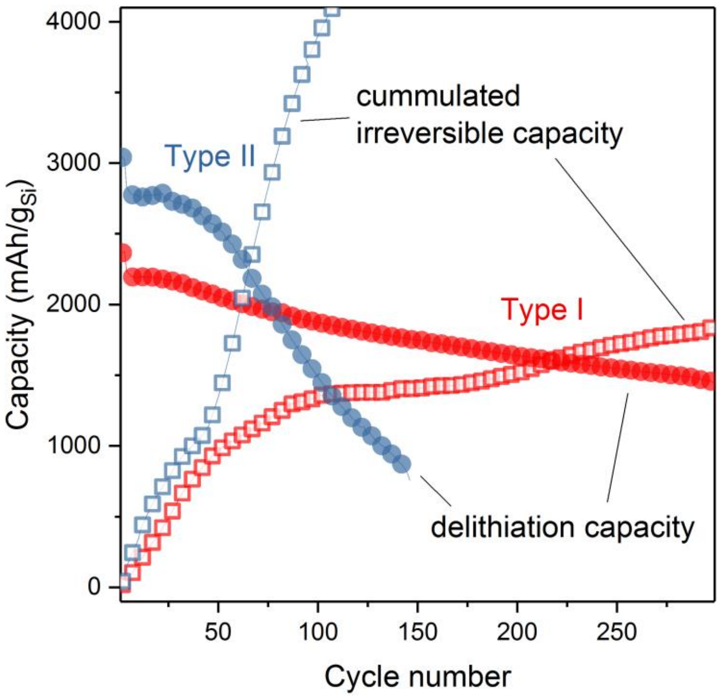

Figure S13. Cumulated irreversible capacity for the two different types. The rate of the build-up of lost capacity goes through at least two phases (changes at around 50 cycles).

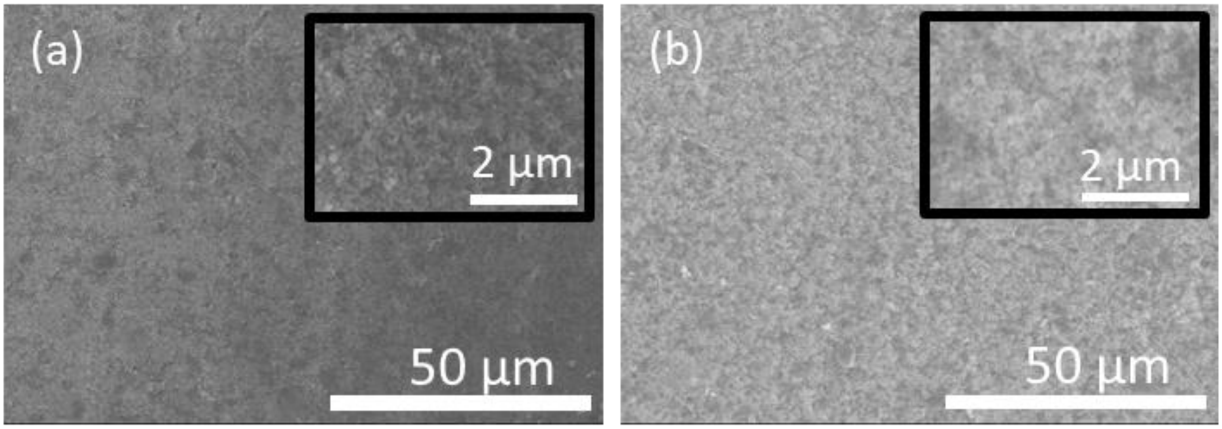

Figure S14. SEM images of the (a) type I and (b) type II electrodes prior to cycling, indicating the pristine, uncracked morphology.

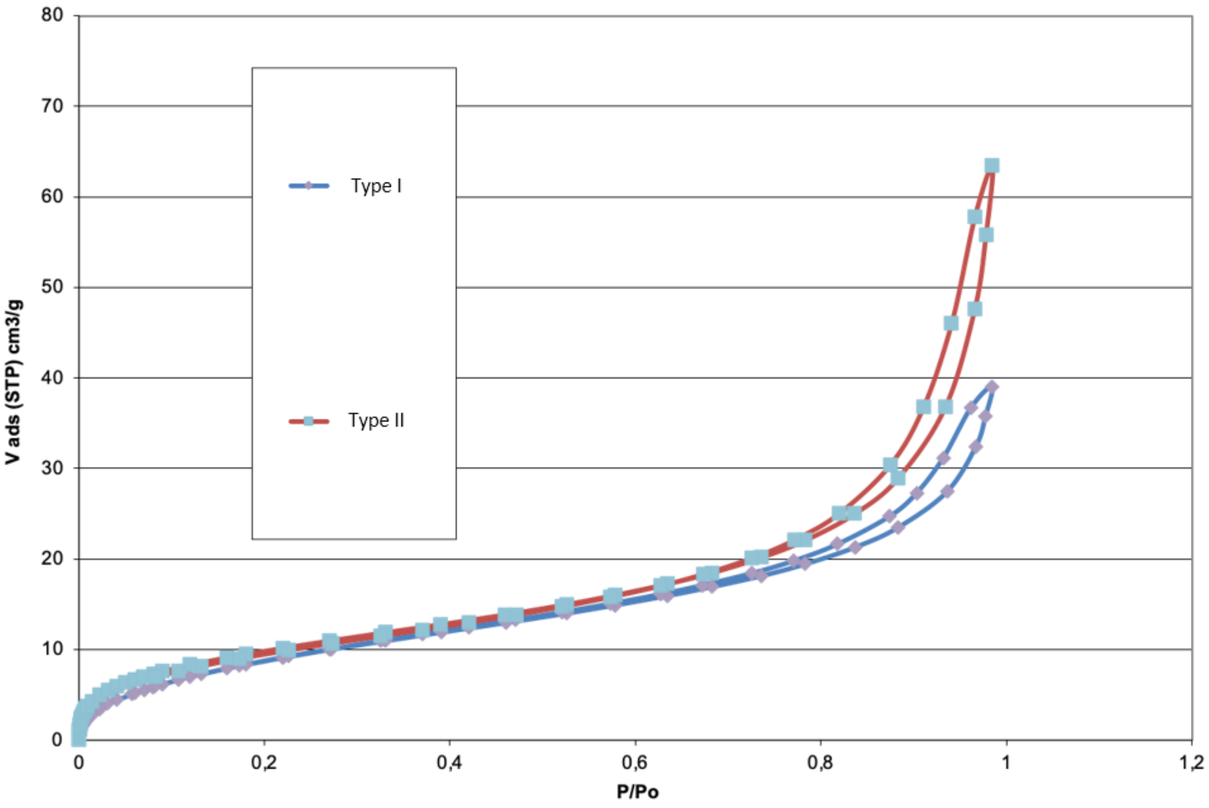

Figure S15. Nitrogen adsorption-desorption isotherms for type I and type II particles.
